# 3D histopathology of stenotic aortic valve cusps using *ex vivo* microfocus computed tomography

**DOI:** 10.3389/fcvm.2023.1129990

**Published:** 2023-04-25

**Authors:** Camille Pestiaux, Grzegorz Pyka, Louise Quirynen, David De Azevedo, Jean-Louis Vanoverschelde, Benoît Lengelé, David Vancraeynest, Christophe Beauloye, Greet Kerckhofs

**Affiliations:** ^1^ Mechatronic, Electrical Energy and Dynamic Systems, Institute of Mechanics, Materials and Civil Engineering, UCLouvain, Louvain-la-Neuve, Belgium; ^2^ Pole of Morphology, Institute of Experimental and Clinical Research, UCLouvain, Brussels, Belgium; ^3^ Pole of Cardiovascular Research, Institute of Experimental and Clinical Research, UCLouvain, Brussels, Belgium; ^4^ Division of Cardiology, University Hospital Saint-Luc, Brussels, Belgium; ^5^ Department of Materials Engineering, KU Leuven, Heverlee, Belgium; ^6^ Prometheus, Division for Skeletal Tissue Engineering, KU Leuven, Leuven, Belgium

**Keywords:** aortic stenosis, microstructural characterization, ex vivo imaging, microfocus computed tomography, 3D histopathology

## Abstract

**Background:**

Calcific aortic stenosis (AS) is the most prevalent heart valve disease in developed countries. The aortic valve cusps progressively thicken and the valve does not open fully due to the presence of calcifications. *In vivo* imaging, usually used for diagnosis, does not allow the visualization of the microstructural changes associated with AS.

**Methods:**

*Ex vivo* high-resolution microfocus computed tomography (microCT) was used to quantitatively describe the microstructure of calcified aortic valve cusps in full 3D. As case study in our work, this quantitative analysis was applied to normal-flow low-gradient severe AS (NF-LG-SAS), for which the medical prognostic is still highly debated in the current literature, and high-gradient severe AS (HG-SAS).

**Results:**

The volume proportion of calcification, the size and number of calcified particles and their density composition was quantified. A new size-based classification considering small-sized particles that are not detected with *in vivo* imaging was defined for macro-, meso- and microscale calcifications. Volume and thickness of aortic valve cusps, including the complete thickness distribution, were also determined. Moreover, changes in the cusp soft tissues were also visualized with microCT and confirmed by scanning electron microscopy images of the same sample. NF-LG-SAS cusps contained lower relative amount of calcifications than HG-SAS. Moreover, the number and size of calcified objects and the volume and thickness of the cusps were also lower in NF-LG-SAS cusps than in HG-SAS.

**Conclusions:**

The application of high-resolution *ex vivo* microCT to stenotic aortic valve cusps provided a quantitative description of the general structure of the cusps and of the calcifications present in the cusp soft tissues. This detailed description could help in the future to better understand the mechanisms of AS.

## 1. Introduction

Calcific aortic stenosis (AS) is the most prevalent heart valve disease in developed countries, as it affects about 29% of the population over 65 (1). It is characterized by the thickening of the aortic valve (AV) and is associated with the growth of calcifications within the extracellular matrix of the valve cusps. The preliminary stage of the disease corresponds to sclerosis, in which the valve is thickened and contains focal areas of calcification while the mobility of the cusps is considered normal. With time, the disease impairs the proper functioning of the valve by decreasing its aperture. This results in stenosis, left ventricular (LV) hypertrophy and finally heart failure. Advanced AS causes clinical symptoms such as decreased exercise tolerance, syncope and dyspnea (2, 3).

Severity of stenosis is evaluated based on *in vivo* parameters such as the mean pressure gradient (MPG) across the AV, the AV area and the peak aortic jet velocity. This is mostly measured using *in vivo* ultrasound imaging, such as transthoracic two-dimensional and Doppler echocardiography. This allows verification of the integrity and motion of the valve and the blood flow parameters, respectively (4, 5). However, several characteristics of AS remain unclear. For instance, the complex mechanism resulting in calcifications is not fully understood yet. Several studies suggest that calcifications arise from a succession of events, involving endothelial damage, lipid infiltration, inflammation, fibrosis and finally mineralization (2, 5–8). However, no preventive treatment has successfully been applied to slow down the AS progression (9, 10). Moreover, the spatial distribution, size and shape of the calcifications have been scarcely and inconsistently described, although they are the predominant feature causing the AV narrowing (2, 11, 12). Refining the pathogenesis of AS and of calcifications might thus open the way for new treatment strategies. In addition to the presence of calcifications, thickening of the cusps is also recognized as a major step in the disease (13). Some rare studies mention the preferential thickening and/or calcification of the non-coronary cusp compared to the other two (7, 14, 15). However, current literature contains few studies about the normal value of the AV thickness and the threshold values for sclerosis and stenosis (14, 16).

To be able to better characterize the microstructure of heart valves (i.e., the complex arrangement of the extracellular matrix components at the microscale and the presence of calcification), especially in case of stenosis, X-ray computed tomography (CT) is a valuable solution. It has already been extensively used *in vivo* (XCT) (17, 18) and *ex vivo* (microCT) (11, 19–21) to image mineralized tissues. In the particular case of calcific AS, it was used to quantify the volume fraction of calcification and to demonstrate the correlation between the aortic valve calcium score and the hemodynamic parameters obtained from Doppler echocardiography both *in vivo* and *ex vivo* (17, 19). However, despite being successfully used to define the severity of AS, with a better accuracy than echocardiography alone (17, 22–24), high-resolution microCT has never been applied to quantitatively compare the microstructural properties and composition of the valve and of the calcifications in different diagnosis groups of AS.

The aim of this study was to obtain a quantitative description of the microstructure and composition of calcified AV cusps using high-resolution microCT, for a better understanding of the calcification mechanism within the AV. In addition to the volume proportion of calcification, which is usually obtained from microCT, we examined the calcifications in terms of size, amount, number of particles and density composition. Changes in the soft tissues of the cusp were also described. Finally, our imaging technique was applied to different clinical diagnoses of AS.

## 2. Materials and methods

### 2.1. Description of AS severity

The severity of AS is evaluated based on echocardiographic parameters. High-gradient severe AS (HG-SAS) corresponds to MPG ≥ 40 mmHg, AV area < 1 cm^2^ and peak aortic jet velocity ≥ 4 m/s while moderate AS is defined by MPG <  40 mmHg, AV area ≥ 1 cm^2^ and peak aortic jet velocity from 2 to 4 m/s (25). However, some intermediate cases exist, as described in Table 1. This study contains three diagnosis groups: HG-SAS, moderate AS, and normal-flow low-gradient severe AS (NF-LG-SAS).

**Table 1:** Classification of AS with reduced AV area opening (≤1 cm^2^) based on parameters measured *in vivo* using ultrasound imaging, according to (2, 26).

		High-gradient (MPG ≥ 40 mmHg)	Low-gradient (MPG < 40 mmHg)
Normal-flow (SVi > 35 ml/m^2^)	Preserved LVEF (> 50%)	High-gradient severe AS (HG-SAS)	Normal-flow low-gradient severe AS (NF-LG-SAS)
Low-flow (SVi < 35 ml/m^2^)	Preserved LVEF (> 50%)	High-gradient severe AS (HG-SAS)	Paradoxical low-flow low-gradient severe AS (PLG-SAS)
	Reduced LVEF (< 50%)	High-gradient severe AS (HG-SAS)	Classical low-flow low-gradient AS

AS, aortic stenosis; LVEF, left ventricular ejection fraction; MPG, mean pressure gradient; SVi, stroke volume index.

### 2.2. Patient selection

Patients were selected, as described in Boulif et al., (23), after a diagnosis of NF-LG-SAS, moderate AS or HG-SAS. AS caused by radiotherapy or irradiation were part of the exclusion criteria. For this retrospective study, only 14 patients for whom the number of samples collected at surgery was equal to three cusps per valve, were included. Among them, 5 were diagnosed as NF-LG-SAS, 2 as moderate AS and 7 as HG-SAS. One non-stenotic aortic valve cusp was included as non-calcified sample. It was provided by the donor bank and not used for transplantation because of a subtle commissural fusion. The study protocol was approved by the local ethical committee (2014/21NOV/560 and 2021/13JAN/014) and all patients gave informed consent prior to inclusion into the study.

### 2.3. High-resolution microfocus X-ray computed tomography imaging

All samples were preserved at −80 °C until sample preparation. They were thawed at room temperature and slightly dried on a paper tissue. Then, they were mounted in a sample holder, including a borosilicate bead to normalize the gray values in the reconstructed images. All samples were imaged using a Phoenix Nanotom M (GE Measurement and Control Solutions, Germany) equipped with a 180 kV/15 W energy nanofocus X-ray tube and a diamond-coated tungsten target. The voxel size was 14 µm. For one NF-LG-SAS cusp, zoom images at two different spatial resolutions were performed after cutting a region of interest from the sample. All acquisition and reconstruction parameters are listed in Table 2.

**Table 2:** Microct acquisition and reconstruction parameters, cusp N4.1 corresponds to the NF-LG-SAS cusp that was imaged at two higher resolutions after the selection of a region of interest.

	All cusps	Cusp N4.1	
Voxel size (µm)	14	5	1.2
Filter material	0.5 mm Al	0.5 mm Al	0.5 mm Al
Source voltage (kV)	90	90	90
Tube current (µA)	400	230	170
Exposure time (ms)	500	500	1,250
Tube focus mode	0	0	1
Number of images	800	1,600	1,600
Average	1	3	3
Skip	0	1	1
Fast scan mode	Yes	No	No
Acquisition time (min)	6’40’’	57’	2 h17’
Beam hardening correction	9	9	9

All microCT datasets were reconstructed with the Datos|x software (GE Measurement and Control Solutions, Germany) and exported as XY slices (.tiff). For one sample, an in-house developed MATLAB (The MathWorks, Massachusetts, USA) script was used to convert the 16-bit slices (.tiff) to 8-bit slices (.bmp), while simultaneously normalizing the histogram range to the dynamic range of the dataset. All the other datasets were normalized to that one using a second in-house developed MATLAB script with the borosilicate bead and the sample holder as reference materials. MATLAB scripts are available on github (27, 28).

### 2.4. Image segmentation and structural analysis of the entire cusp

For each dataset, the valvular tissue was segmented from the background using Avizo (Thermo Fisher Scientific, Bordeaux, France). Briefly, a region-of-interest was defined to remove the sample holder, and the cusp was then binarized using a manually selected threshold (25–255). For this, CTAn was used (Bruker MicroCT, Kontich, Belgium). Then, a closing step was performed to remove the small holes (square of size 5) and 3D analysis was performed (i.e., volume and thickness distribution, 3D analysis module) on the entire cusp (including both soft tissues and calcifications).

### 2.5. Image segmentation and structural analysis of calcifications

Within the entire cusp, the calcifications were segmented using a multilevel Otsu segmentation (3 levels, CTAn). To have the same threshold values for all samples, the mean from the multi-level segmentation was selected (i.e., 75 ± 4.05, 139 ± 6.11 and 197 ± 5.94 for low, moderate and high density, respectively) and applied to all samples. The volume fraction of all calcifications within the cusp (all densities included, gray values from 75 to 255) was quantified (CTAn). All voxels identified as calcification (no distinction of density) and connected to each other were defined as one calcified particle (labeling module from Avizo). All particles of 10 voxels or less were not considered. The number of calcified particles and their volume were then computed. Three categories of volume were defined. The first category corresponds to particles up to 1 × 10^−3^ mm^3^, the second one includes particles from 1 × 10^−3^ mm^3^ to 1 mm^3^ and the third category contains particles from 1 mm^3^ and above. Then, to compensate the partial volume effect (PVE) observed when segmenting the calcifications with different densities, an in-house protocol was developed (CTAn). First, the high-density calcifications (HD, gray values 197–255) were segmented. This volume was dilated by two voxels and removed from the entire cusp. Then, the moderate and low-density calcifications were successively segmented (gray values 139–255 and 75–255 respectively). Additionally, an opening step (square of size two) was performed on both selections (MD and LD). Finally, each of the three selections (HD, MD and LD) were individually dilated by one voxel to compensate the volume loss and the final volumes were quantified for each density. This PVE correction resulted in a final volume loss of 11.02% ± 1.47% (Supplementary Figure S1), but eliminated wrongly assigned edge voxels of calcifications to another density class. 2D and 3D renderings were performed using Avizo.

### 2.6. Scanning electron microscopy (SEM) imaging

The region of interest cut from sample N4.1, and previously imaged at higher resolution with microCT, was embedded in paraffin in a home-made cylindrical shape holder. It was then cut with a micro-precision saw (Accutom 50, Struers LLC, USA) at the specific location determined based on the high-resolution microCT data. After mounting on a SEM holder, the top surface was sputtered with gold to make the sample conductive. The images were generated with the backscattered electron detector on the Ultra 55 FEG SEM (Zeiss, Germany) at a voltage of 15 kV.

### 2.7. Statistical analysis

GraphPad Prism 9 (GraphPad Software, California, USA) was used for the statistical analysis and data visualization. To compare the NF-LG-SAS and HG-SAS groups, Mann-Whitney tests were performed for each structural parameter. Depending on the normality of the datasets, correlations were evaluated with Pearson or Spearman tests. To assess the effect of the diagnosis and of the density on the volume quantified for each density calcification, a multiple 2-way ANOVA test was performed. *p*-values below 0.05 were considered to be significant; *: *p* < 0.05, **: *p* < 0.01, ***: *p* < 0.001 and ****: *p* < 0.0001. In the bar graphs, the mean value of the different samples is indicated by the height of the bars. Error bars represent the standard deviation and the individual datapoints are given.

## 3. Results

### 3.1. *Ex vivo* microCT provides the 3D spatial localization of calcifications and reveals that the volume fraction of calcification is significantly lower in case of NF-LG-SAS than in HG-SAS

**Figure 1:**
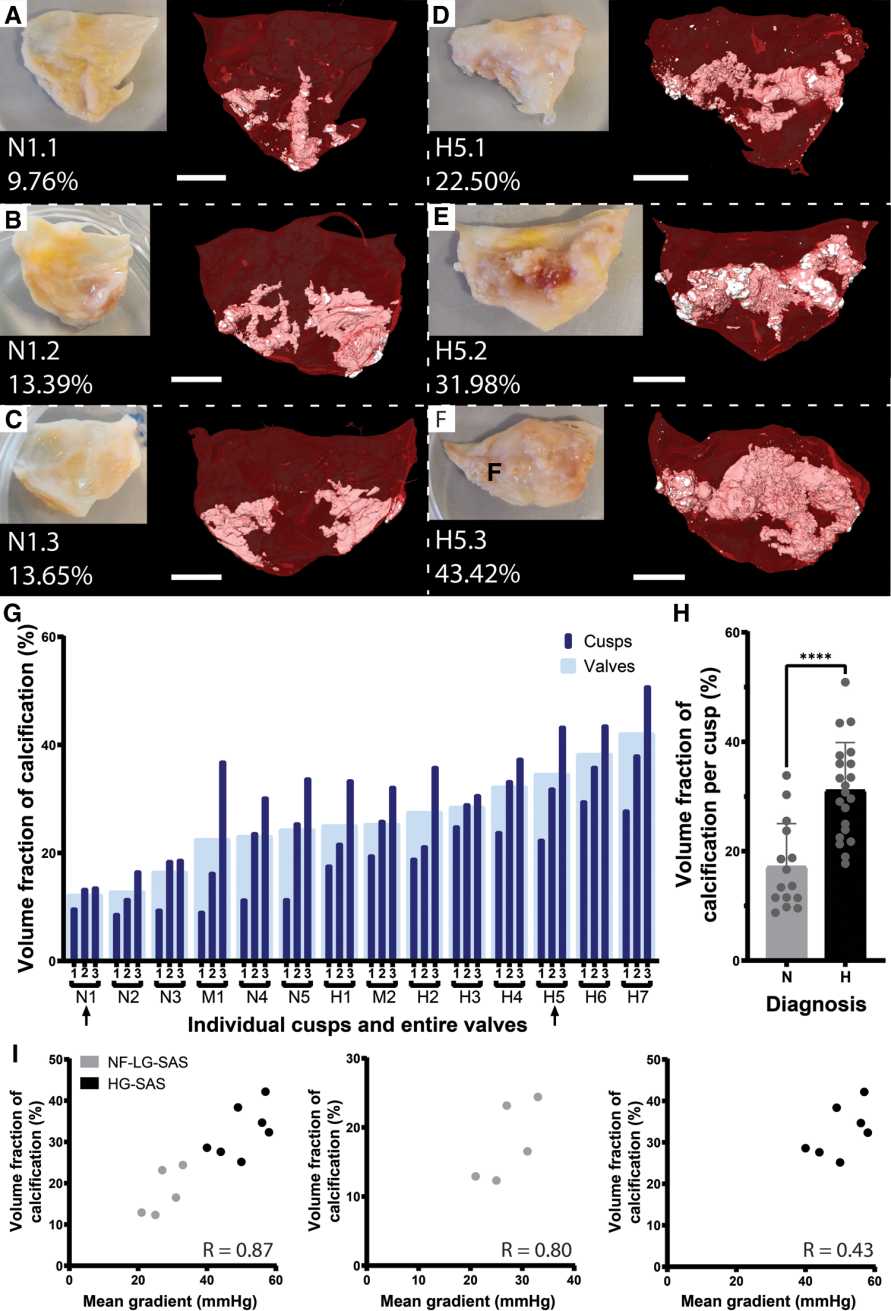
Quantification of the volume fraction of calcification per cusp and per valve. (**A–F**) Photographs and microCT-based 3D renderings of the three cusps for patient N1, diagnosed with NF-LG-SAS (**A–C**) and patient H5, diagnosed with HG-SAS (**D–F**). For the 3D renderings, soft tissue is shown in red and calcification in white. Scale bars = 5 mm. (**G**) Volume fraction of calcification for each cusp (dark blue) and for the entire valve (light blue). Arrows indicate the samples illustrated in (**A–F**). (**H**) Bar graph comparing the mean volume fraction of calcification for the NF-LG-SAS and HG-SAS groups. Individual datapoints are given for each cusp (*n* = 15 and *n* = 21 per group respectively). (**I**) Correlation between the volume fraction of calcification and the mean pressure gradient across the aortic valve for all patients (left), NF-LG-SAS patients (center) and HG-SAS patients (right). N, NF-LG-SAS; M, moderate AS; H, HG-SAS, ****: *p*-value < 0.0001.

The volume fraction of calcification present in the soft tissues of calcified AV cusps explanted from human patients was computed both per valve and per cusp, based on *ex vivo* microCT images (Figure 1). First, we were capable of assessing the spatial distribution of the calcifications. Although calcifications are sometimes described as extrinsic (11), the 3D visualization obtained in our work demonstrated that calcifications were located inside the cusp soft tissues, with some rare particles visible on the subendothelial surface. They were mainly located on the adherent edges of the cusp, along the attachment to the anulus, while for the most calcified samples, not only the edges of the cusp were calcified, but the midportion of the cusp, between free and adherent edges, was affected as well (Figures 1A–F). Then, the volume fraction of calcification was assessed. It ranged from 9.8% to 50.9%, which demonstrates the wide variation among patients, but also among the three cusps of the same patient (Figure 1G). The volume fraction was significantly lower for the NF-LG-SAS cusps than for the HG-SAS ones (Figure 1H). The volume fraction of calcification was correlated with the mean pressure gradient across the AV. This correlation was stronger when both NF-LG-SAS and HG-SAS groups were considered compared to each group taken individually (Figure 1I). While the severity of NF-LG-SAS is still under discussion, in this case, most of the samples corresponding to NF-LG-SAS had a lower relative amount of calcification than the moderate AS samples. Since only 2 patients (corresponding to 6 cusps) had a moderate AS diagnosis, they were excluded from the statistical analyses.

### 3.2. The number and size of the particles constituting the calcifications is related to the severity of AS

**Figure 2:**
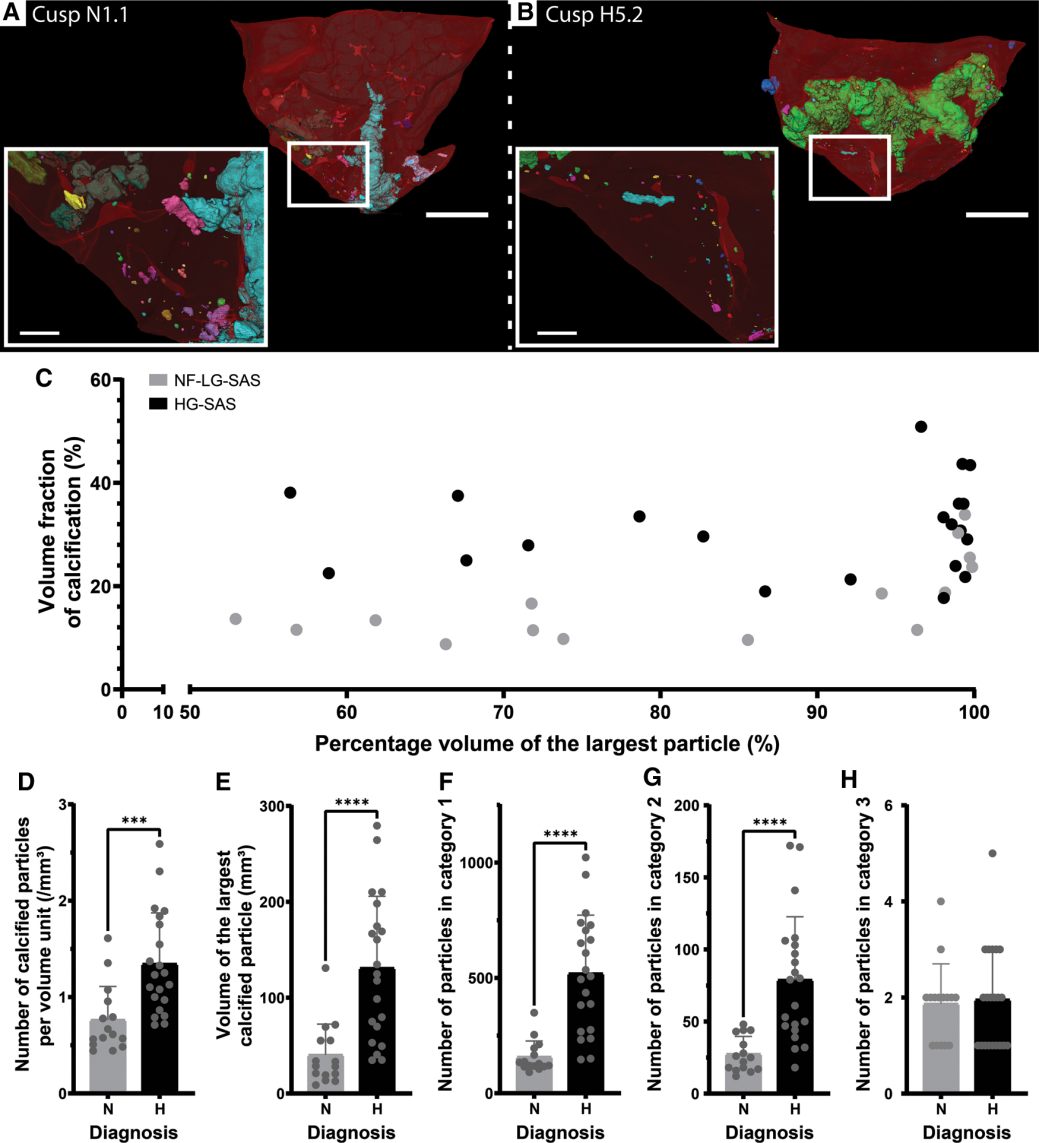
Quantification of the number and volume of calcified particles. (**A,B**) 3D renderings of the cusp N1.1 (**A**) and cusp H5.2 (**B**). Clusters of voxels identified as calcification and connected to each other are considered as one particle and displayed in the same color. Colors of particles are independent of any morphometrical quantification. Soft tissue is shown in red. A magnification (white square) is shown in the inset. Scale bars = 5 mm for the overview images and 1 mm for the inset. (**C**) Percentage volume of the largest particle in function of the volume fraction of calcification present in the cusp for the NF-LG-SAS samples and the HG-SAS samples. (**D–H**) Bar graphs comparing the number of calcified particles per volume unit (**D**), the volume of the largest particle (**E**), the number of particles in category 1 (**F**), category 2 (**G**) and category 3 (**H**), respectively for the NF-LG-SAS and HG-SAS groups. N, NF-LG-SAS; H, HG-SAS, ***: *p*-value < 0.001, ****: *p*-value < 0.0001.

Apart from a volumetric assessment of the calcifications, this study aimed at structurally describing the calcifications present in the cusp in an effort to investigate calcification formation mechanism. A calcified particle was defined as a cluster of voxels previously identified as calcification and connected to each other (Figures 2A,B). A significantly higher number of particles per volume unit were present in case of HG-SAS than in NF-LG-SAS (Figure 2D). Although the absolute volume of the largest particle in HG-SAS cusps was significantly higher than in NF-LG-SAS cusps (Figure 2E), for all cusps, it represented more than 50% of the calcification volume present in the sample (Figure 2C). The classification of the particles in three categories according to their size demonstrated that the increased number per volume unit observed in HG-SAS was mainly due to the presence of many small-sized particles (Figures 2F–H). Indeed, NF-LG-SAS cusps and HG-SAS cusps contained on average 157 and 519 particles, respectively, in the first category (corresponding to particles up to 1 × 10^−3^ mm^3^). In the second category (from 1 × 10^−3^ mm^3^ to 1 mm^3^), these numbers dropped to 27 and 79, respectively, and the third category (from 1 mm^3^ and above) only contained on average two particles for both NF-LG-SAS and HG-SAS diagnoses (Figures 2F–H). Consequently, both the number of particles per volume unit and the size of the largest particle were increased in case of HG-SAS. Moreover, the difference in the number of particles mainly relied on the quantity of the smallest ones, while the largest particles were equally present for both diagnoses.

### 3.3. *Ex vivo* microCT allows to distinguish and quantify different densities in the calcifications, but they did not differ between the different diagnosis groups

**Figure 3:**
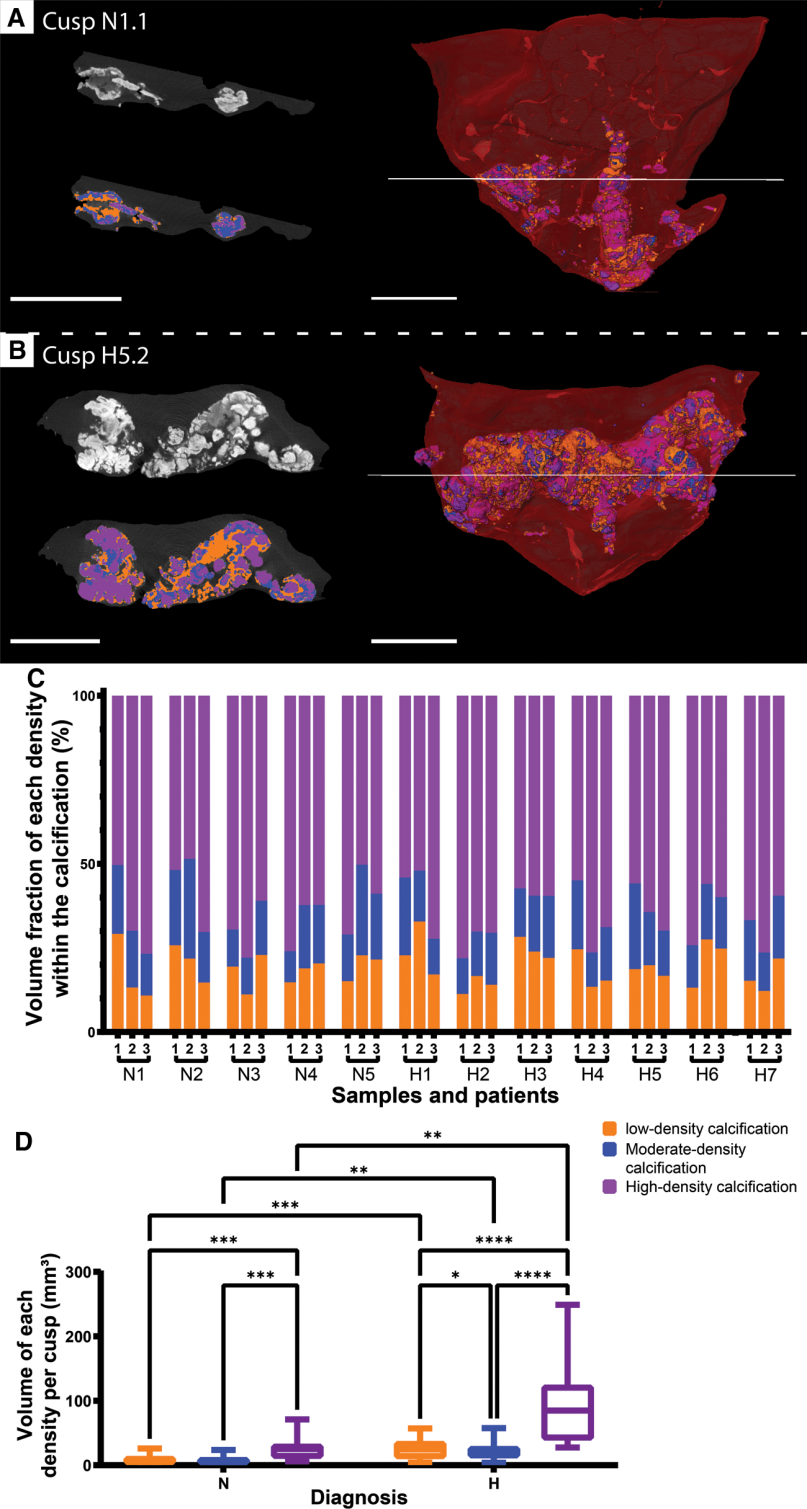
Distinction of three levels of density within the calcifications. (**A,B**) Ortho-slices in gray scale (top left) and after segmentation (bottom left) and 3D renderings (right) for cusp N1.1 (**A**) and cusp H5.2 (**B**). Low-density is shown in orange, moderate-density is shown in blue and high-density is shown in purple. Soft tissue is shown in dark gray on ortho-slices and in red on 3D renderings. Scale bars = 5 mm. (**C**) Volume fraction of each density group within the calcification for every cusp. (**D**) Box plots comparing the impact of the diagnosis within a density group, as well as of the density within a diagnosis group on the absolute volume of each density group. *: *p*-value < 0.05, **: *p*-value < 0.01, ***: *p*-value < 0.001, ****: *p*-value < 0.0001, N, NF-LG-SAS; H, HG-SAS.

In addition to the number of calcified particles, we also investigated the density composition of the calcifications. The visualization of the calcifications in normalized gray scale allowed the distinction of different densities that were divided into three categories corresponding to low, moderate and high density (Figure 3). The spatial distribution of densities within the calcifications was highly heterogeneous for both NF-LG-SAS and HG-SAS diagnoses (Figures 3A,B and Supplementary Movie S1). The high-density calcifications had the highest relative volume in all cusps, while low- and moderate-densities were less, but equally, present (Figure 3C). The volume fraction of each density group could not be used to discern the NF-LG-SAS from the HG-SAS diagnosis groups. Both the diagnosis and the density groups had a strong effect on the absolute volume quantified for each density group (Figure 3D).

### 3.4. Cusp volume and thickness correlate with the degree of calcification

**Figure 4:**
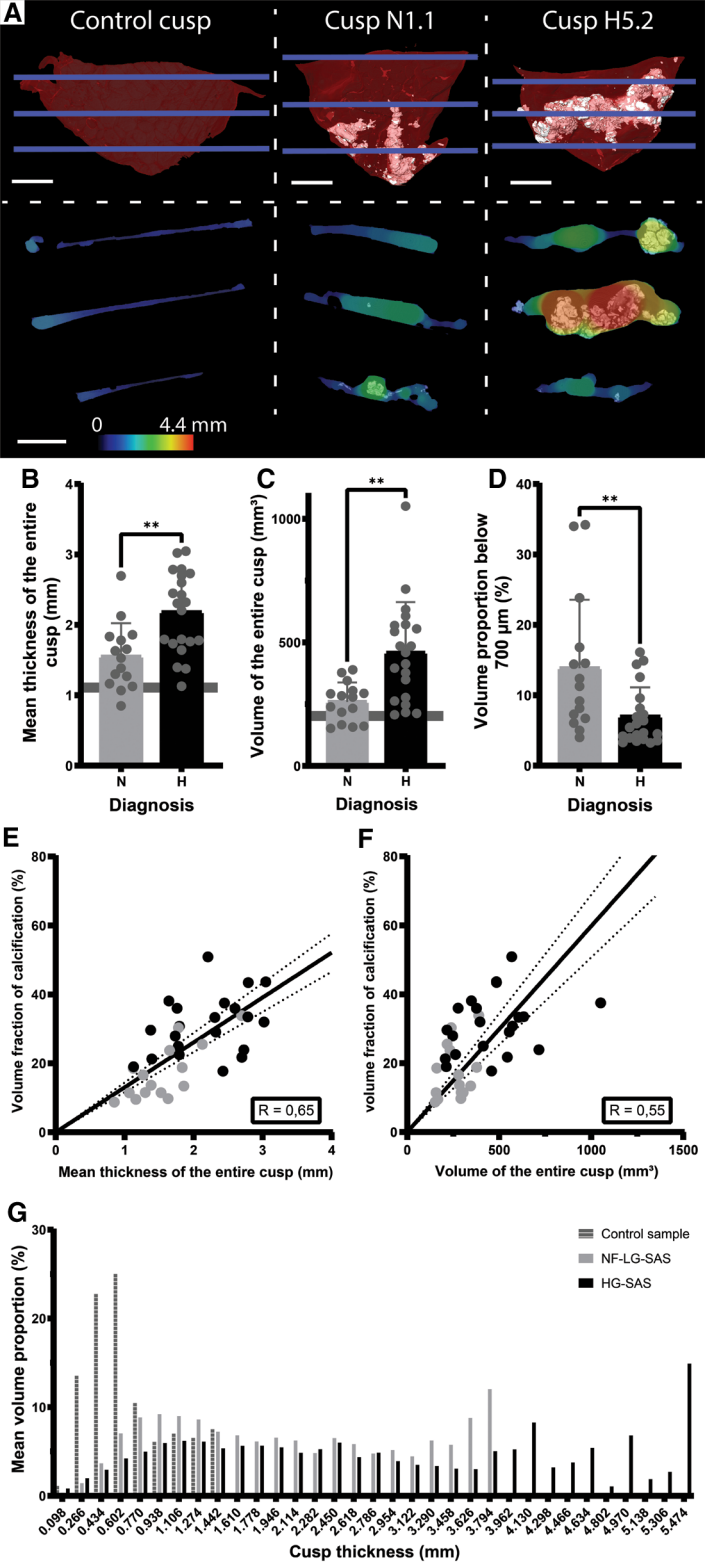
Quantification of the volume and thickness of the entire cusp. (**A**) 3D renderings (top) of the control cusp, sample N1.1 and H5.2 and ortho-slices (bottom) showing simultaneously the calcifications and the thickness values at three heights corresponding to the blue lines in the 3D renderings. For the 3D renderings, soft tissue is shown in red and calcification in white. Scale bars = 5 mm. Thickness is represented by the color scale bar. (**B–D**) Bar graphs comparing the mean thickness of the entire cusp (**B**), the volume of the entire cusp (**C**) and the volume proportion of the cusp with a thickness below 700 µm (**D**) for the NF-LG-SAS and HG-SAS groups. The horizontal line indicates the value for the control cusp, not shown on D for clarity (corresponding to 64.6%). (**E,F**) Correlations between the volume fraction of calcification and the mean thickness of the entire cusp (**E**) and the volume of the entire cusp (**F**), respectively. Dotted lines indicate the 95% confidence bands. (**G**) Average histogram of the thickness distribution in terms of volume proportion for the control cusp, the NF-LG-SAS and HG-SAS cusps. N, NF-LG-SAS; H, HG-SAS, **: *p*-value < 0.01.

Calcific AS is not only characterized by the growth of calcifications within the cusp of the AV, but it is also associated with valve thickening and an increased cusp volume (Figure 4). The simultaneous visualization of the calcifications and the thickness of the cusps revealed that the thickest area of the cusp corresponds to the large calcifications (Figure 4A and Supplementary Movie S2). While the mean thickness of the NF-LG-SAS and the HG-SAS cusps was about 1.6 mm and 2.2 mm respectively, the mean thickness of the non-calcified cusp was about 0.7 mm. The presence of a higher amount of calcification, such as in HG-SAS samples, resulted in a significant increase of the cusp thickness and volume, compared to the NF-LG-SAS group (Figures 4B,C). Moreover, the volume proportion of the cusp that still has a thickness corresponding to a healthy valve, defined by the mean thickness of the non-calcified cusp (0.7 mm), was significantly different between NF-LG-SAS and HG-SAS diagnoses. On average, 13.9% of the cusp volume from NF-LG-SAS samples and 7.0% from HG-SAS samples still had a normal thickness (< 0,7 mm). The volume proportion of the non-calcified cusp with a thickness below 0.7 mm was equal to 64.6% (Figure 4D). This suggests that the cusp continues to thicken as the mineralization progresses. Both the thickness and volume were correlated with the volume fraction of calcification present in the cusp (Figures 4E,F). Unlike gross evaluation-based studies, imaging the samples in 3D at high spatial resolution provided the full distribution of the thickness along the cusp (Figure 4G), allowing the observation of local variations.

### 3.5. *Ex* vivo high-resolution imaging revealed micro-sized calcifications

**Figure 5:**
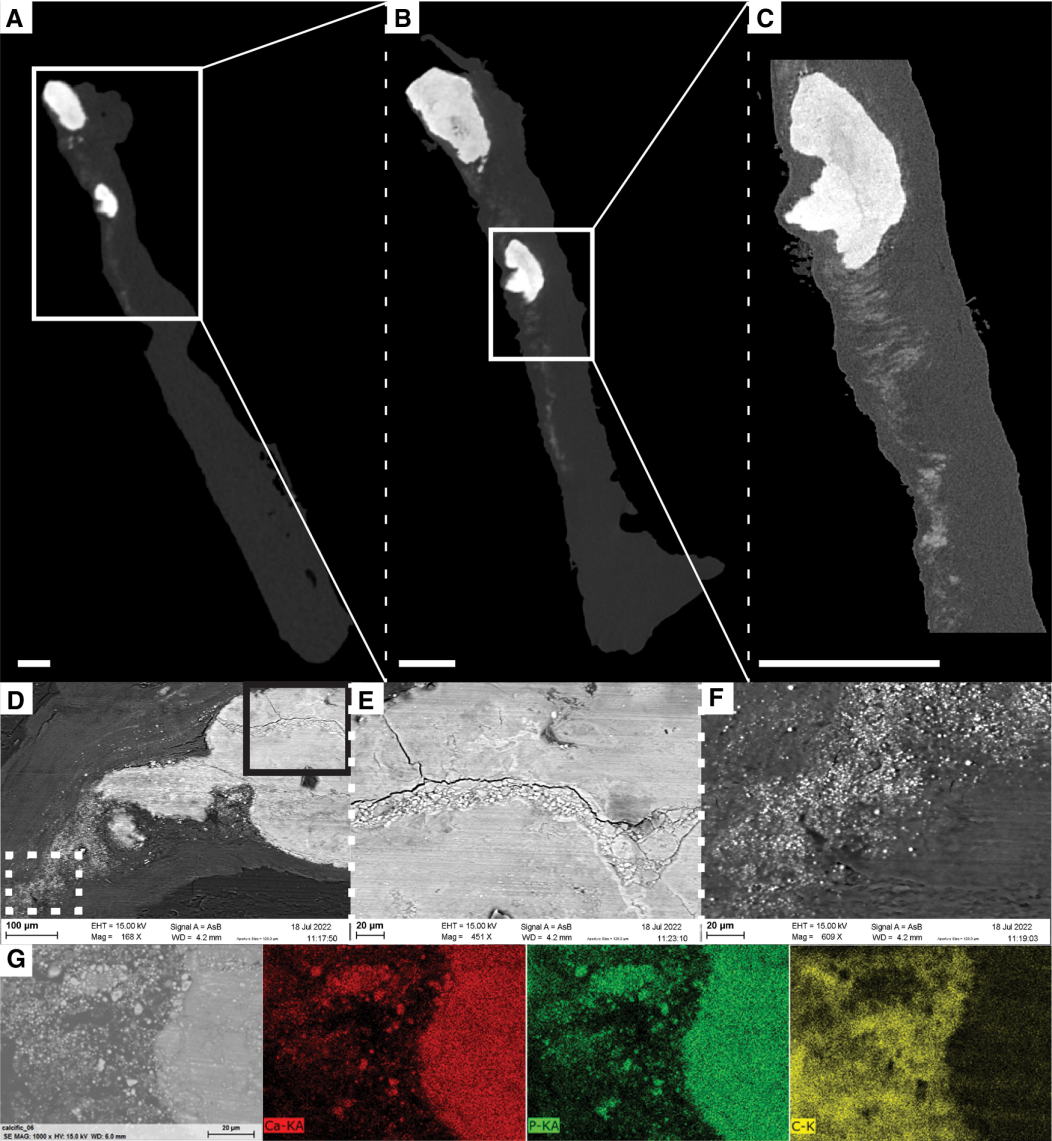
Visualization of soft tissue changes next to dense calcifications in sample N4.1. (**A–D**) Ortho-slices from *ex vivo* microCT obtained with different voxel sizes: 14 µm (**A**), 5 µm (**B**) and 1.2 µm (**C**). Scale bars = 0.5 mm. White squares correspond to the location of magnification. (**D–F**) SEM images with accumulation of particles within a dense calcification (**E**) and visualization of micro-sized particles in the soft tissue (**F**) obtained with backscattered electron detector, and different magnifications: x168 (**D**), x451 (**E**), and x609 (**F**). Solid black square and dotted white square in D correspond to the location of magnifications in the dense calcification and in soft tissues, respectively. (**G**) EDX analysis obtained at the border between the soft tissue and the dense calcification demonstrating that dense and micro-sized calcifications are composed of Calcium and Phosphate, while soft tissue is composed of Carbon.

Another indication of pathology was also visualized by changes in the gray values of the soft tissues surrounding dense calcifications (Figure 5). Although being recognized as more attenuating areas, they were not identified as calcifications due to the limited spatial resolution and the partial volume effect (Figure 5A). Higher resolution imaging performed with microCT provided more accurate visualization of the dense calcification and of surrounding tissues, but it did not allow to distinguish individual micro-sized particles (Figures 5B,C). Only the SEM examination revealed that the more attenuating areas actually correspond to clusters of small calcification islets in the vicinity of dense calcifications (Figures 5D,F and Supplementary Movie S3). The composition analysis obtained from EDX proved that main and micro-sized calcifications were composed of calcium and phosphate while carbon was located in the surrounding soft tissues (Figure 5G). The micro-sized particles present in the soft tissues were mainly visualized in the least calcified samples. In addition to the observations made within the soft tissues, SEM also allowed to visualize the accumulation of small particles within dense calcifications (Figure 5E).

## 4. Discussion

**Figure 6:**
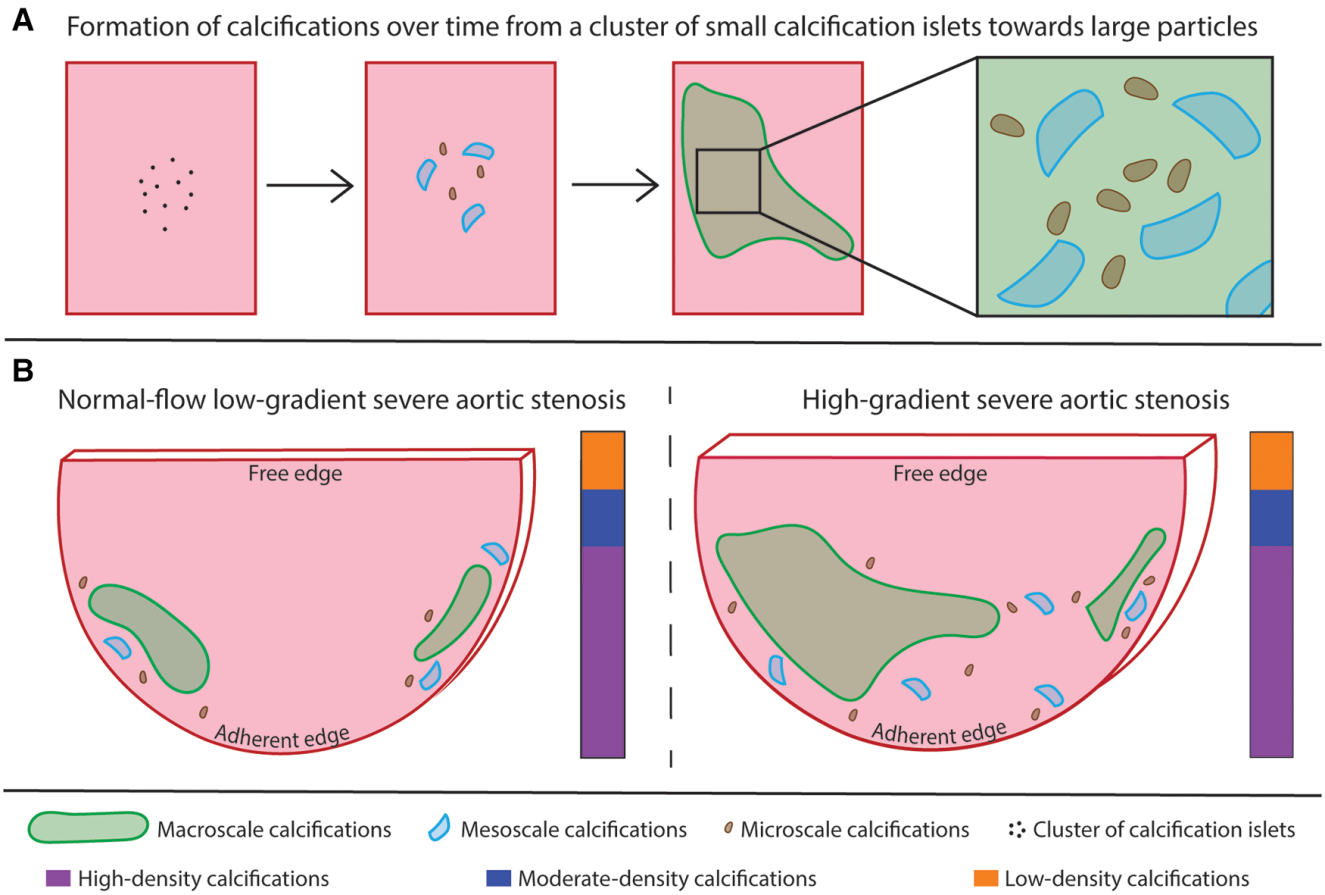
Summary scheme. (**A**) illustration of the hypothesis about the evolution of a cluster of small calcification islets into dense calcifications over time. Large calcifications correspond to the accumulation of small particles. (**B**) comparison of the two patterns of calcification. The calcifications are present in higher relative amount and the cusp is thicker in the HG-SAS group than in NF-LG-SAS. In the less calcified samples, the calcifications are mainly located at the adherent edges of the cusp, while for the most calcified samples, the midportion of the cusp is affected as well. The absolute volume of the largest calcified particle is higher in the HG-SAS group than in NF-LG-SAS, but it corresponds to at least 50% of the total calcification volume in all cases. The macroscale particles are present in equal number in both groups while the meso- and microscale particles are both present in higher number in the HG-SAS groups than in NF-LG-SAS. The calcification density is evenly heterogeneous in both diagnosis groups (illustrated by the vertical bar).

In this study, a quantitative structural description of both the entire cusp and the calcifications was obtained using *ex vivo* microCT. The clear visualization of the cusp and the distinction of calcifications from the soft tissues allowed the quantification of several structural parameters, such as the volume fraction of calcification, the number and volume of calcified particles, and the distinction and quantification of the amount of different densities within the calcification in full 3D. Moreover, the volume and thickness of the entire cusp could be quantified, and changes in the soft tissues were observed, demonstrating the alteration of the extracellular matrix of the cusp. The observations and hypotheses of this study have been illustrated in a summary scheme (Figure 6). This structural characterization has the capability to provide a better description of the microstructural changes due to AS.

*In vivo* computed tomography and *ex vivo* microCT are two well-known X-ray-based techniques to visualize mineralized tissues (24, 29). *In vivo,* the degree of AV calcification is usually used in atypical cases of aortic stenosis (if hemodynamic and anatomic parameters are controversial). It is evaluated by the Agatston score (based on density weighting) or, less frequently, the calcium volume score (based on the volume quantification) (30). Both electron-beam computed tomography (EBCT) and multidetector computed tomography (MDCT) have been used to quantify AV calcification *in vivo.* Although EBCT was the first modality to have a sufficient temporal resolution to image the heart, MDCT, which was developed more recently, has a better signal-to-noise ratio and spatial resolution (31–33). These are two important parameters allowing the exclusion of non-valvular calcifications in the examination. In comparison with the microCT method presented in this study, *in vivo* techniques are used with a totally different purpose of diagnosis and quantification of the degree of calcification to assess the need for heart valve replacement. Although *ex vivo* microCT does not provide radiodensity measures in standardized units (Hounsfield units used in clinical CT), which requires advanced calibration of the equipment, it produces high-resolution images. In comparison with *in vivo* CT approaches, for which the voxel's largest dimension corresponds to the 3mm-slice thickness, in this study, microCT has an isotropic voxel size of 14 µm. It enables the extraction of detailed microstructural information of explanted samples that could not be collected without organ removal and high radiation dose. *Ex vivo* microCT could be used in a multiscale manner in combination with for example MDCT to provide complementary data and better characterize AV calcifications.

E*x vivo* microCT is thus an already described tool that provides a detailed visualization of the AV cusps and an accurate quantification of the amount of calcification present in the soft tissues in case of calcified AS (11, 19–21). Nevertheless, the mean volume of calcification obtained in our study, in average 316 mm^3^ per valve, is lower than the value published previously, which was 600 mm^3^ (19). This difference might be explained by the amount of tissue collected during surgery and by different imaging strategies: (i) imaging per cusp in our case and the valve as a whole in the previous one, (ii) use of different voxel size (14 µm and 76 µm, respectively for our study and the study of Chitsaz et al.).

Our results demonstrated that it was possible not only to quantify the volume fraction of calcifications, but also to describe their 3D spatial distribution. Although the spatial distribution of the calcifications among the cusp does not correspond to a clear pattern, most of the calcification cores have an attachment to the adherent edge of the cusp, along the insertion to the aortic wall, while the free edge is usually free of calcification. This is especially visible in the less calcified samples and confirms what was reported before (6, 11). As the calcification increases, the midportion of the cusp becomes affected as well. Although Orzechowska et al*.* reported that minerals were mainly present on the surface of the cusp and corresponded to extrinsic mineralization, our data clearly shows that calcifications are mostly located inside the cusp (11).

We showed a variation in the volume fraction of calcification among the three cusps from a single valve, which suggests that they are affected differently by the disease. This confirms the results from Raman spectroscopy previously performed on one valve (15). Current literature often describes one cusp that would be especially more calcified than the other two, identified as the non-coronary cusp (7, 14, 15). The preferential growth of calcifications in one of the histological layers, identified as the fibrosa layers, located on the aortic side of the cusp, has also been mentioned (2, 7, 8). Nevertheless, such identification was not possible in our study because the true anatomy of the cusps and their *in vivo* position was not indicated during excision. Interestingly, both the non-coronary cusp, which is deprived of coronary artery, and the aortic side of the cusp are thought to be prone to decreased shear stress compared to the other two cusps and to the ventricular side of AV, respectively. Shear stress is therefore a determinant parameter in the initiation of AS. It is described as a continuous stimulus for the endothelium that should inhibit pathological changes such as calcifications, however without direct proof (2, 7, 34). The distinction of the three cusps and their orientation (i.e ventricular and aortic sides) could help to validate and to better understand the role of shear stress. We therefore recommend to use a systematic annotation of the cusps during surgery for future studies, which could easily overcome this limitation. The application of the detailed analysis presented in this study to bicuspid aortic valves might provide more information regarding the role of the shear stress in the calcification mechanism. Moreover, to fully characterize the process of calcification growth, it would have been necessary to include samples at an earlier stage of the disease.

Using high resolution *ex vivo* microCT, the volume of calcifications can be quantified, but they can also be clearly visualized. Unlike *in vivo* and/or low-resolution imaging, the technique presented here permits the observation of small-sized calcified particles, which are a key element to understand the formation of larger size calcifications. Indeed, small-sized calcified particles can be interpreted as onsets of calcifications. Our results showed that all AS samples contain one or two main calcified particles and that some onsets of calcifications are present in a variable amount. As the calcification increases, both the volume of the main calcified particles and the number of onsets of calcifications rise. This quantitative structural analysis could reveal different phases of AS. From this perspective, the first pattern (one or two relatively small main calcified particles and some onsets of calcifications) defines a quiescent form of valvular disease and the second one (one or two large calcified particles and many onsets of calcifications) corresponds to an active form of AS. In the second case, the numerous onsets of calcifications are expected to evolve quickly into larger calcified particles, which would further hinder the opening and closing of the valve and, consequently, the proper functioning of the heart. The distinction of such onsets of calcification could reveal the initial location of calcifications and help to determine the link between calcification growth, blood flow and shear stress. The inability of *in vivo* imaging to identify the small-sized particles, although they are present in high number and correspond to onsets of calcifications, highlights the need for high resolution *ex vivo* imaging when investigating the calcification mechanisms resulting in AS. The presence of calcified particles of different sizes in AS cusps was previously described by Razzolini and colleagues. As they performed a macroscopic evaluation, the size threshold used was 4 mm. Calcified particles were named microaggregates (below 4 mm) or macroaggregates (above 4 mm) (12). According to our results, we propose a new nomenclature based on *ex vivo* high-resolution imaging such as microCT. Macroscale calcifications are defined by a volume of 1 mm^3^ or above, microscale calcifications have a volume below or equal to 1 × 10^−3^ mm^3^ and, in between these two categories, calcified objects are qualified as mesoscale calcifications with a volume from 1 × 10^−3^ to 1 mm^3^. Our examination reveals that both the volume of the largest macroscale calcification and the number of micro- and mesoscale calcifications are increased when the proportion of calcification rises.

In addition to the number and size of calcified particles, different normalized gray values were also distinguished within the calcifications. The distinction of different densities within the calcifications was demonstrated in a previous study (11). However, previous studies did not assess the spatial distribution of densities in 3D and did not link it to the clinical diagnosis of the pathology. In our study, the examination of the spatial distribution for each density group confirms the heterogeneity observed by Orzechowska et al. However, it does not demonstrate that small-sized calcifications are particularly less dense than the larger ones or whether they would evolve into high-density calcifications. Cusps with a less advanced stage of AS should also be analyzed to better understand the presence of different densities within the calcifications. We foresee potential applications in animal models, such as the Reversa mice model (35), allowing the analysis at different time points.

To investigate the changes in thickness of the cusps due to AS, *in vivo* ultrasound imaging is mostly used (14) or *ex vivo* gross evaluation is performed (36). However, the absolute volume of the individual cusps is rarely assessed due to technical limitations. The 3D imaging technique that we present here provided quantitative values for the volume and the mean thickness of the cusp. Moreover, the full thickness distribution, including local variations, could also be quantified. It allowed us to observe that the thickened areas correspond to the location of large calcifications. As thickening is recognized as a precursor sign of AS, local thickening of the cusp might be an indication for future calcifications (13, 14). Moreover, we also assessed the thickness distribution of a non-calcified cusp, whereas this information is scarcely available in the current literature. Studying the volume and thickness distributions of both healthy and calcified cusps would provide a better description of the native structure of the valve and would help to determine precise thresholds for pathological variations. Since thickening is an indication for tissue remodeling, this quantification might also be used to detect an abnormality within the soft tissues and to indicate a region-of-interest for further investigations.

In addition to the change in thickness and volume of the cusp, we were also capable of visualizing changes in the soft tissues of the AV cusp with the presence of more-attenuating areas corresponding to micro-sized particles in the extracellular matrix. Initial visualization obtained with microCT allowed to locate a region of interest that could be investigated at higher resolution with microCT and, as demonstrated on one sample in our case, with other imaging modalities, such as SEM. Although microCT did not allow to visualize individual particles, it was used to indicate where the sample should be cut to be imaged with SEM. SEM images revealed that the previously defined region of interest contained small calcification islets located next to dense calcifications. This area would correspond to the location of future dense calcifications. Two different processes have been previously described to explain the formation of calcifications (2, 37). First, the osteogenic process is initiated by the differentiation of valvular cells into osteoblast-like cells. In this case, a highly organized bone-like mineral matrix is the basis for the nucleation of minerals. The second process is an apoptotic process of the valvular interstitial cells that is promoted by several stimuli and results in apoptotic cell aggregates. They consist of amorphous deposits of calcium and phosphorus crystals. The visualization of extracellular matrix changes such as the ones shown in our results, before the formation of dense calcifications, could help to better describe these two processes and to clarify their role in the calcification formation. Moreover, assessing the microstructure of extracellular components around these onsets of calcification could provide a detailed description of the remodeling associated with cusp thickening and calcification growth, such as excessive production and disorganization of collagen fibers (2, 7).

As case study in our work, the quantitative structural analysis we performed was applied to two patient groups: NF-LG-SAS and HG-SAS to determine whether they were structurally different from each other and to evaluate whether *ex vivo* microCT could be used to better understand the particular case of NF-LG-SAS diagnosis. The medical prognosis of NF-LG-SAS is still under discussion and it is still not clear whether NF-LG-SAS is a truly severe AS, as calcified as HG-SAS or whether it is an intermediate stage of AS in which calcifications will still increase and result in high pressure gradient (26, 38–42). The summary scheme outlines the main differences observed between the two diagnosis groups (Figure 6B). First, our quantification of the calcification volume demonstrated that NF-LG-SAS cusps were less calcified than the HG-SAS ones. In case of HG-SAS, the main calcified particle was significantly larger than that of NF-LG-SAS and the onsets of calcifications were more numerous, thus corresponding to an active form of AS. However, due to the low number of samples included in our study, it was not possible to determine whether the diagnosis alone could explain the different patterns of calcification (one or two relatively small main particles and few onsets of calcification vs. few large main particles and many onsets of calcifications) or whether the two patterns could be observed in each patient group. The quantification of the volume and spatial distribution of the different densities observed within the calcification did not permit to distinguish specific characteristics for NF-LG-SAS compared to HG-SAS. The thickness and volume of NF-LG-SAS cusps were significantly lower than those of HG-SAS. Consequently, all the structural parameters quantified in our work support several previously published studies that evaluate NF-LG-SAS as a less advanced AS progression than other severe AS cases (39, 42–46). Since AS classification contains several controversial subgroups, the imaging approach described in this study could be used to better characterize and understand each AS phenotype.

The technique presented in this study presents some limitations. The need for *ex vivo* imaging to obtain high-resolution images prevents the possibility for time-lapsed follow-up of the calcified cusps. This would help to investigate the evolution of the amount and spatial distribution of calcifications, the evolution of the calcified particles, as well as the different densities within the calcifications. It could also validate that changes observed in the extracellular matrix of the cusp correspond to future dense calcifications. Moreover, tissue shrinkage was observed on sample N4.1, which underwent longer scan time (more than 3 h in total). This limitation could be easily overcome by using an environmental stage to control the scanning atmosphere. Although our study allowed to distinguish different densities within the calcification based on normalized grey values, it was not possible to determine true density values. This would require the use of reference materials with known density. As mentioned previously, our conclusions were also limited by the number of cusps and the absence of cusps affected by an earlier stage of AS.

As the technique presented here is non-destructive, it can be combined with other imaging modalities to investigate the components of the extracellular matrix. For instance, it was used as a preliminary imaging step to indicate where to cut the sample for SEM analysis. It could also be done prior to classical 2D histology, which could help to describe the endothelial damage, lipid infiltration, inflammation and fibrosis preceding mineralization. It was not possible in this study to combine both SEM imaging and classical 2D histology after microCT because the sample was embedded in paraffin in a home-made cylindrical sample holder and covered with gold. As a future perspective, we are convinced that combining *ex vivo* microCT with high-atomic number contrast-enhancing staining agents (CESAs), corresponding to contrast-enhanced computed tomography or CECT, could allow the visualization of the different histological layers in the cusp. This technique can also be applied in cryogenic conditions, corresponding to cryo-CECT, which was successfully used to perform histopathological analysis of entire mouse hearts (28). Such 3D imaging techniques have the potential to better define the microstructure of the extracellular matrix of healthy aortic valve and the disorganization induced by AS. Unlike classical 2D histology, they do not require any demineralization step. It could for example validate the preferential growth of calcifications within the fibrosa layer or the presence of neovascularization in case of AS (2, 47). It could also be used to quantify the excessive production of collagen fibers associated with the cusp thickening.

## 5. Conclusion

In conclusion, we used high resolution *ex vivo* microCT to provide a new quantitative description of the general structure of calcified cusps in case of AS and of the calcifications present in the cusp soft tissues. In the future, the results presented here can be used to further improve *in vivo* imaging protocols and the interpretation of clinical data. The detailed description of the stenotic AV cusps provided in our results could also help to better understand the mechanism of AS.

## Data Availability

The raw data supporting the conclusions of this article will be made available by the authors, without undue reservation.

## Supplementary material

SUPPLEMENTARY FIGURE 1

Partial volume effect (PVE) correction for each density group. A–B: ortho-slices of sample N1.1 showing the classification of calcification into low, moderate and high-density before (A) and after (B) the PVE correction. C: absolute volume of each density group within the calcification for every cusp. Two bars are given for each cusp, before PVE correction (left) and after PVE correction (right).

SUPPLEMENTARY MOVIE S1

Distinction of three levels of density within the calcifications. Ortho-slices (right) and 3D renderings (left) of cusps N1.1 and H5.2. The horizontal white lines present in the 3D renderings correspond to the location of the ortho-slices. Soft tissue is shown in dark gray on ortho-slices and in red in the 3D renderings.

SUPPLEMENTARY MOVIE S2

Thickness distribution in 3D in cusps N1.1 and H5.2. Soft tissue is shown in red and calcification in white. The thickness distribution is shown on the ortho-slice progressively cutting the 3D renderings and is represented by the color scale bar.

SUPPLEMENTARY MOVIE S3

Visualization of micro-sized calcifications on microCT ortho-slices successively obtained at a voxel size of 14 µm, 5 µm and 1.2 µm. In the 3D renderings, the location of the ortho-slice is shown by the orange line, soft tissue is shown in red and calcification in white.
